# Sound Detection Monitoring Tool in CNC Milling Sounds by K-Means Clustering Algorithm

**DOI:** 10.3390/s21134288

**Published:** 2021-06-23

**Authors:** Cheng-Yu Peng, Ully Raihany, Shu-Wei Kuo, Yen-Zuo Chen

**Affiliations:** 1 Department of Electronic Engineering, National Chin-Yi University of Technology, Taichung 41170, Taiwan; 4a813033@gm.student.ncut.edu.tw (U.R.); s4a913019@student.ncut.edu.tw (Y.-Z.C.); 2 Department of Electrical Engineering, National Taipei University of Technology, Taipei 10608, Taiwan; t108318008@ntut.org.tw

**Keywords:** sound detection, monitoring, CNC milling machine, fast Fourier transform, K-means

## Abstract

Computer numerical control (CNC) is a machine used in the manufacturing industry to produce components quickly for the engineering field or the desired shape. In the milling process carried out by CNC machines, sometimes vibrations occur that cause unwanted cracks or damage, which if left unchecked, will cause more severe damage. For this reason, this study describes how to monitor and analyze the sound produced by CNC during the milling process. This study uses six sound sample videos from YouTube, and there are two modes: (1) the operating mode is three different shapes with *XY*, *XZ*, and *XYZ* axes, and the second (2) is based on material differences. Namely, wood, Styrofoam, and plastic. The sound generated from all samples of the CNC milling processes will be detected using a sound detection program that has been designed in the LabVIEW using a simple microphone. The resulting sound frequency will be analyzed using the fast Fourier transform (FFT) process in spectral measurements, which will produce the amplitude and frequency of the detected sound in real time in the form of a graph. All frequency results that have been obtained from the sound detection monitoring tool in the CNC milling machine will be imported into the K-means clustering algorithm where the different frequencies between the resonant frequency and noise will be classified. Based on the experiments conducted, the sound detection program can detect sounds with a significant level of sensitivity.

## 1. Introduction

Computer numerical control (CNC) [1] is a machine that is universally used in the industrial world to facilitate the manufacture of goods according to the desired shape and condition by entering commands that have been designed. The working principle of CNC begins with the sequence of shapes to be made by typing directly on a CNC machine or a PC pad with CNC programming applications. The program or order that has been made after that is sent and executed by a CNC machine carries out the order process according to the program and the desired shape.

During the CNC milling process, the milling machine will emit sounds and vibrations [2], where these vibrations produce a specific frequency range according to the pressure applied and also as an indicator of the smoothness of the milling process, it can be determined based on the vibration during the process, whether this is normal or has the possibility of errors such as balance, physical, or mechanical determination. 

A. A. Jaber et al. [3] provide signal considerations as a technique that uses engine condition data as a solution to avoid sudden shutdowns and to prevent failures in complex systems. By using wavelet transforms in the LabVIEW program and MATLAB capabilities, the faults in industrial machinery can be detected in the early stages before they have reached critical levels. This author [4] presents a way to detect a given echo audio signal from human voice or sonar based on knowledge of acoustics and signal processing using sound detection designed in LabVIEW program produced in the form of a graphical algorithm for real-time echo signal detection. P. Tanuska et al. [5] implement an anomaly detection and prediction for an assembly process maintenance system to the bearing wheel bearings using sound signal analysis, which is evaluated with time-domain statistical indicators and frequency analysis, where identification of bearing wheel damage is carried out to prevent unexpected stoppages. 

For this reason, the purpose of this simulation is to monitor the milling process while the machine is running by simulating a sound detection [6] program in the LabVIEW software [7]. This program uses a microphone [8] to capture the sound of the milling process and transmit it to the program, where the results of the simulation are made in the form of images and frequency graphs, and amplitude and applying fast Fourier transform (FFT) [9] for the analysis of spectral measurements will be clustered by a K-means algorithm [10,11]. This author [12] compares batch K-means processing with K-means streaming processing based on value, cost distance, and cluster distribution factors to analyze the attributes of batch K-means processing and K-means streaming processing and also to find the limitations of both processing models. This study [13] describes the design, development, and implementation of a method for photocardiograph signal detection. The human heart and lung signals are detected using a simple microphone via a personal computer; signals were recorded and analyzed using LabVIEW software. Automatic classification of normal and abnormal heart sounds, murmurs, and lungs. Voices are presented using fast Fourier transform analysis.

This author [14] provides an analysis of the sound field emitted by selected CNC machine tools. Acoustic holography for the three-axes DMC 635eco machine tool and the five-axes vertical machine center DMU 65 Monoblock measures source identification and noise level. The acoustic holographic method allows for identification and measurement. The results of this study are presented in the form of pictures and diagrams. This author [15] presents an analysis of the noise emitted by selected machine tools in a production room (under industrial conditions). Identification of noise sources and levels was carried out using the UNIT 352 measurement system for DMU 50, BGO-CNC/RV/R, FU 251, FW 801, FWC25/H. Effective noise reduction is very significant from the perspective of minimizing noise at various work stations. The test results conclude with detailed recommendations for CNC machine tool operators to use hearing protection while working.

This research [16] consists of semantic object detection using You Only Look Once version 3 (YOLOv3) and object extraction through photo separation based on the improved K-means algorithm. Determination of K values sourced from semantic data and depth data allows the K-means algorithm to ensure the number of segmentations that match the actual scene. This approach improves the segmentation capabilities of photos in real-world panoramas. Experiments using open source information sets demonstrate this. The average processing time and segmentation accuracy of the improved K-means algorithm is 20, 36% faster and 3.12% greater than the conventional K-means algorithm accuracy. The proposed procedure is 6.69% greater than the SuperCut extraction procedure. R. Wu et al. [17] used the improved K-means clustering method to group feature motions and to separate large dots and obtain accurate background feature points for motion monitoring. In this study, we also used K-means as the clustering of all frequency results.

This monitoring is carried out to supervise the running process of the CNC milling, whether it is running well or there is a disturbance so that the operator can find out about it through monitoring. In this study, before the experiment was carried out on CNC samples from YouTube, the calibration was carried out to prove that the program was running well by simulating the tone sound “DO RE MI FA SOL”, which had been tried 10 times randomly and produced a fixed and stable frequency in the frequency range. So that proves that the program is made stably.

After ensuring that the calibration results and sound detection are stable, an experiment was carried out on six samples of CNC Milling videos. With sound detection that has been designed in LabVIEW, the output of sound detection in LabVIEW is in the form of amplitude and frequency graphs, the results of which are exported using Origin Software and the resulting high-quality graphics. The results of the resonant signal frequency from sound detection that have been carried out will be classified with a K-means algorithm to separate the resonant frequency signal or noise so that it makes classification easier; the results of this K-means clustering will be plotted again with Excel to obtain a good picture.

## 2. Proposed System and Methodology for Sound Detection Monitoring Tool in CNC Milling Sounds

### 2.1. Block Diagram of Sound Detection Monitoring and Analysis System

**Figure 1:**
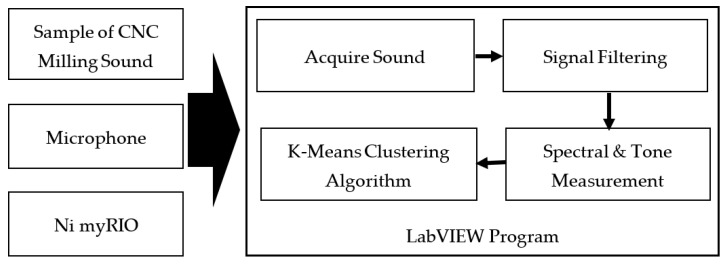
Block diagram of sound detection.

Figure 1 shows the experimental system and implementation of the experiment in the form of a block diagram. The sound detection from the CNC milling is captured by the microphone to be programmed and will be classified using the clustering algorithm by K-means.

### 2.2. Instrument Design and Experimental Setup

#### 2.2.1. Sound Detection for Hardware Acquisition

**Figure 2:**
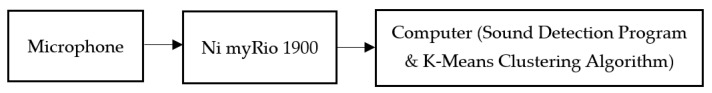
System structure of hardware.

The integration system uses a sound detection program designed in LabVIEW to obtain the resonant frequency of each sound produced during the milling process on a CNC machine, Figure 2 shown the system structure of hardware for experimental setup.

- Microphone

A microphone is a receiver that translates sound vibrations in the air into electronic signals or writes them onto a recording medium. This study uses Intopic JAZZ-016, which is a mini-PC desktop microphone to complement the computer used for voice recording and a sensitive microphone for the noise problem to make less noise from the environment.

- myRIO (National Instrument)

LabVIEW myRIO 1900 [18] is an embedded system for real-time evaluation. It is a tool to support work including data acquisition and frequency resonance analysis to be more efficient and faster when used on systems that require a fast response such as CNC machines.

#### 2.2.2. Sound Detection for Software Design

LabVIEW provides helpful graphical programming to visualize an application’s aspect, including hardware applications, data measurement, and debugging. It uses a graphic or block diagram-based programming language. The LabVIEW program is known as Vi or Virtual Instruments because its appearance and operation can resemble an instrument. In this study, sound detection that has been made in the LabVIEW program uses the following parameters are shown in Table 1.

#### 2.2.3. Flowchart Diagram Implementation

**Figure 3:**
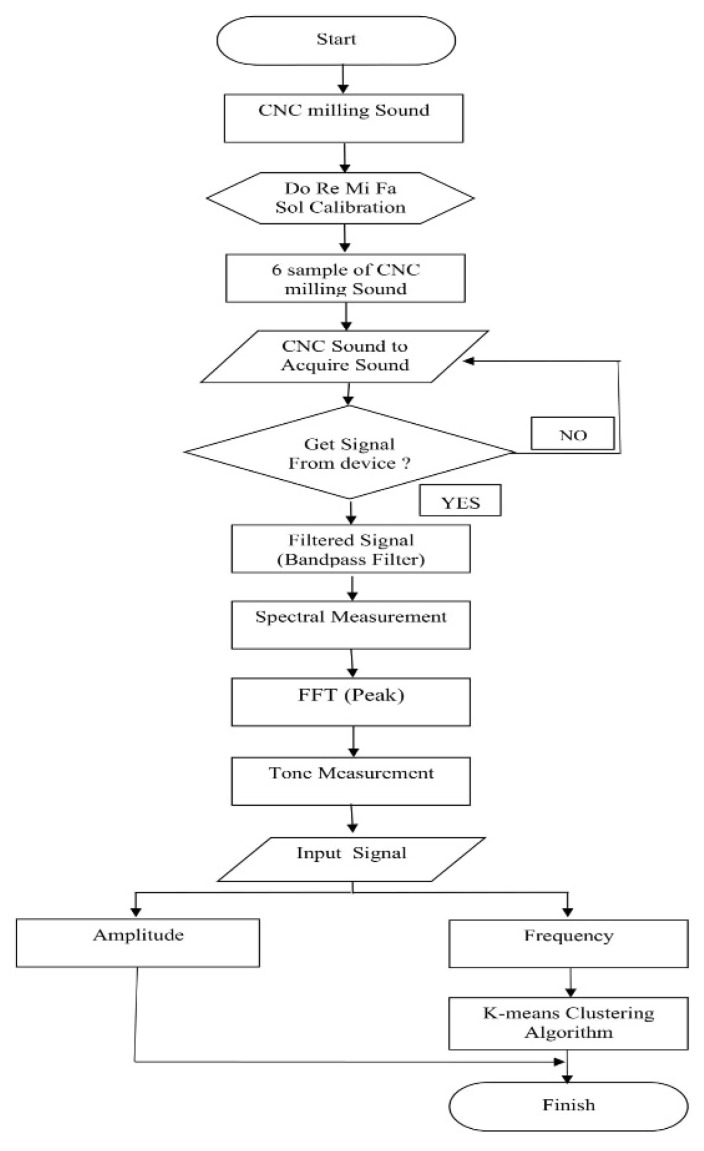
Flowchart diagram.

The experimental process is shown in the flow diagram in Figure 3. In this experiment, a sound sample from a CNC machine process is rotated and captured by a microphone, producing a digital signal in the output sound. Sound is filtered using a bandpass filter with Butterworth topology and analyzed the resonant frequency with FFT in spectral measurements. Once the results are obtained, the frequency will be classified using the clustering algorithm by K-means.

### 2.3. CNC Milling Sounds by Signal Process

#### 2.3.1. Signal Filtering by Butterworth Filter

This study uses sound detection with bandpass as a filter; a bandpass filter passes signals with a frequency range (passband) and pastes signals with frequencies outside this range. For the response filter, the infinite impulse response (IIR) filter is used comprehensively because of the sharper transition area [20]. The filter used is a butterwort topology [21] because the advantages of the Butterworth filter are smooth, monotonous, maximum flatness, with an ideal unity response in the passband and zero in the stopband, and the frequency drops by 3 dB, which corresponds to a specified cutoff frequency.

#### 2.3.2. Spectral Measurement

Spectral measurements are carried out with the resulting spectral magnitude measured in peak values at the resonant frequency. In this study, a zero-phase spectrum was arranged with an average linear value. Tone measurements [22] will be analyzed to determine the single tone with the highest amplitude or find a frequency range.

#### 2.3.3. Resonant Frequency and Amplitude by Fast Fourier Transform

Fast Fourier transform [23] is an algorithm used to represent internal signals discrete-time domain and frequency domain. Discussing FFT-IFFT indeed cannot be separated from discrete Fourier transform (DFT) [24]. DFT is a mathematical transformation method for discrete time signals into the domain frequency. DFT is a mathematical signal transformation method of discrete time, while FFT is the algorithm used to perform the transformation formulated together. Mathematically, DFT can be formulated as follows:

(1) $$X\left[k\right]=\sum_{0}^{N}\;x\;\left[n\right]\;,\;\;\;\;\;\;\;\;W_{N}^{nk}\;\;\;\;\;;\;\;\;k=0,1,2,\ldots N-1\;$$

where$\;W_{N}^{nk}$ is called the twiddle factor and has a value, $e^{\frac{-j2\pi nk}{N}}$, so that

(2) $$X\left[k\right]=\sum_{n=0}^{N-1}\;x\;\left[n\right]\;,\;\;\;\;e^{-}{}^{\frac{j2\pi nk}{N}}\;\;\;;\;\;\;\;k=0,1,2,\ldots N-1$$

Meanwhile, inverse discrete Fourier transform (IDFT) can be formulated as follows:

(3) $$x\left[n\right]=\frac{1}{N}\sum_{n=0}^{N-1}\;x\;\left[k\right],\;\;\;\;\;\;\;\;W_{N}^{-nk}\;\;\;;\;n=0,1,2,\ldots N-1$$

So that the IDFT equation can also be written as follows:

(4) $$x\left[n\right]=\frac{1}{N}\sum_{n=0}^{N-1}\;x\;\left[k\right],\;\;\;\;\;\;\;e^{\frac{j2\pi nk}{N}}\;\;\;;\;n=0,1,2,\ldots N-1$$

FFT is used to reduce the complexity of the transformations performed with DFT.

#### 2.3.4. Clustering Algorithm by K-Means

K-means clustering is a non-hierarchical cluster analysis procedure to partition existing objects into separate, one, or more clusters or teams of objects according to their respective characteristics. Each object with the same characteristics is grouped into the same cluster, and objects with different characteristics are grouped into another cluster. The K-means clustering method seeks to classify the information contained into several groups. Information in one group has the same characteristics and has different characteristics from information in other groups [25].

The average algorithm is one of the simplest and most famous unsupervised learning methods. It is an extension of the vector quantization method in signal processing. With different analysis methods, different cluster classification algorithms are naturally derived, which are generated based on different foundations. The grouping definition of will also changes accordingly.

The aggregation or splitting is selected for screening, and then, an appropriate number of clusters is selected from the result as the final result. The mathematical formula of the average algorithm is as follows:

1. J = objective function;
2. kk_k_ = number of clusters;
3. n = number of cases;
4. X = case *i*;
5. C = centroid for cluster *j*;
6. $\left\|X_{i}^{\left(j\right)}-C_{j}\right\|$ = Euclidean distance between $X_{i}^{}$ and $C_{j}$.

(5) $$\sqrt{\sum_{j=1}^{k}{\left(X_{i}-C_{j}\right)}^{2}}$$

where $X_{i}\;\mathrm{and}\;C_{j}$ are two parts in dimensional Euclidean space [18].

(6) $$J=\sum_{j=1}^{k}\sum_{i=1}^{n}\|X_{i}^{\left(j\right)}-C_{j}\|{}^{2}$$

Algorithmic steps for k-means clustering: X={x1,x2,x3,……xn} are the set of data points, and C={c1,c2,c3,……,cn} are the set of centers. For the first, randomly select “j” cluster centers and calculate the distance between each data point and cluster center, then assign the data point to the cluster center whose distance from the cluster center is the minimum of all the cluster centers. Recalculate the new cluster center using:

(7) $$J_{i}i=\left(\frac{1}{C_{i}}\right)\overset{C_{i}}{\underset{j=1}{\sum }}Xi$$

The K-means average algorithm is to set the number of clusters to be grouped at the beginning and, then, achieve the purpose of grouping by repeatedly modifying until the group center is stable and there is no change. K-means averaging algorithm is based on the sum of the squared difference of the distance between each point in each cluster and the cluster center to which it belongs, as small as possible.

**Figure 4:**
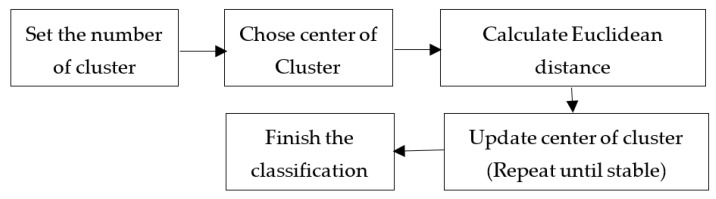
K-means grouping step diagram.

By K-means, the average algorithm has the characteristics of fast calculation speed and real-time update of the group center. It analyzes, classifies, and identifies CNC milling machines’ frequency with different drilling methods and other materials. Figure 4 shows the process of K-means grouping step diagram.

**Figure 5:**
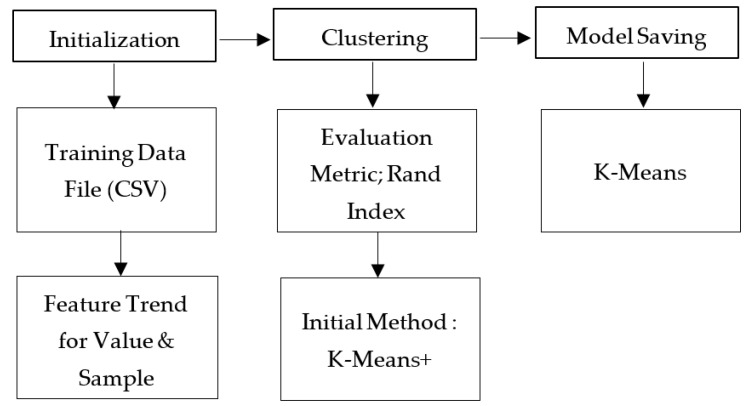
Block diagram of K-means program.

In this study, all experimental results from sound detection samples on the CNC milling process video are carried out 10 times. All frequency data results are collected, and clustering will be run on the K-means program that has been designed on the LabVIEW software. Figure 5 shows the block diagram of the program flow analysis process presenting the input information of initialization, the performance evaluation of clustering, and the sound model saving of milling process that has been designed on the K-means program.

The explanation of the block diagram is explained as follows:

- Initialization

The first thing to do for initialization is to import data through a training data file (CSV) where comma-separated values (CSV) [26] are plain text files that store table and spreadsheet information in the form of table text, numbers, or dates. CSV-designed programs are used to import data from the experimental results of the CNC milling process with a feature trend for value and sample.

- Clustering

After the data are received, they will enter the clustering section using the evaluation metric, the Rand Index is selected to evaluate the clustering model being used, because the lower the metric value, the better the separation of the clustering model. The data will be entered, and the clustering result will be displayed. Additionally, the initial method determines the method for selecting the initial centroid, and K-means ++ (default) is selected to select the initial centroid in a way that always accelerates convergence.

- Model Saving

For the saving model, K-mean is chosen as the model to be saved.

## 3. Experiments

**Figure 6:**
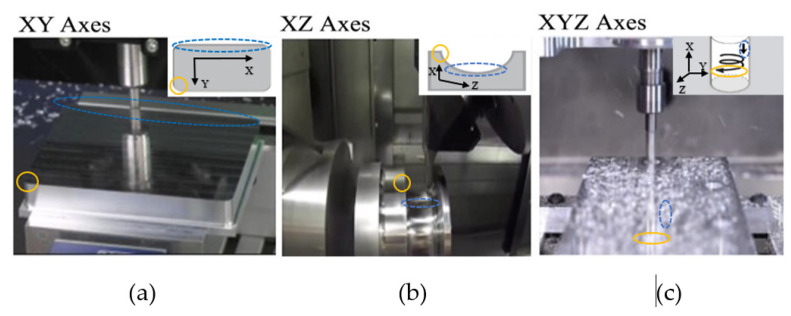
Operation Shapes of (**a**). *XY* axes [31] accessed on 10 December 2019. (**b**) *XZ* axes [32] accessed on 10 December 2019. (**c**) *XYZ* Axes [33] accessed on 5 August 2019.

**Figure 7:**
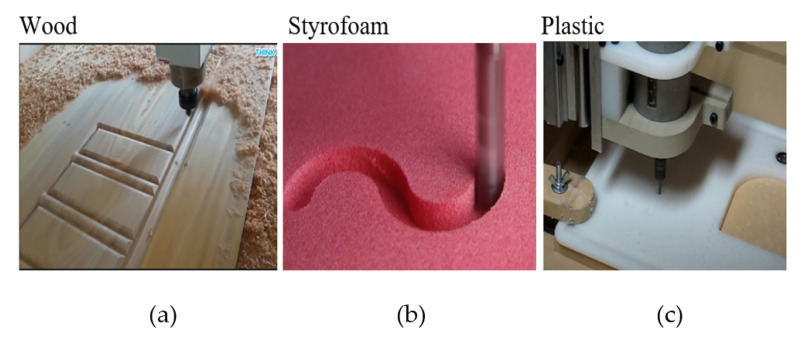
Operation Materials of (**a**) CNC milling process on wood [37] accessed on 10 December 2019. (**b**) CNC milling process on Styrofoam [38] accessed on 10 December 2019. (**c**) CNC milling process on plastic [39] accessed on 10 December 2019.

In this experiment, there are two different modes. The first is the mode of operation shape of the process with *XY*, *XZ*, and *XYZ* axes [27] are shown in Figure 6. The second is an analysis based on the same axes but with different materials Shown in Figure 7, where the materials used are Styrofoam [28], wood [29], and plastic [30]. The 6 video samples of the CNC milling process were obtained from YouTube with the analysis range setting at 1000–1700 Hz.

### 3.1. Working Structure: Operation Shapes of CNC Milling Process

- Movement plane at *XY* axes to obtain corner and edge information.

The *X*-axes and the *Y*-axes form the *XY* coordinate plane and contain points whose triple sequence is (*x*, *y*, 0). The equation = 0 represents the *XY* plane. The horizontal is the *X*-axes as well as the vertical is the *Y*-axes [34]. In Figure 6a, the shape of the material to be sculpted is a rectangle whose motion has an *X* coordinate and a *Y* coordinate. For the CNC machine used on the *XY* axes, this takes a video of the CNC process of the CNC machine from the DATRON MXCube.

- Movement plane at *XZ* axes to obtain the low and high information.

The x-axes and the z-axes form the *XZ* coordinate plane and contain points whose ordered triples are of the form (*x*, 0, *z*). The equation *y* = 0 represents the *XZ* plane [35]. Figure 6b shows that the sculpted shape is half a circle whose motion has an *X* coordinate and a *Z* coordinate.

- Movement plane at *XYZ* axes to obtain inward loop information.

The horizontal line where the wall to the left and the floor intersects is the *X*-axes. The *Y*-axes are where the horizontal line on the wall to the right and the floor intersect. The *Z*-axes are vertical lines on intersecting walls [35,36]. The part of the line from inside the room is the positive part of each axes. This is illustrated by the halves of each axes labeled by *X*, *Y*, and *Z*. In Figure 6c, the sculpture forms a cylinder downward along the *X*, *Y*, and *Z*-axes. For CNC, the cutting machine parameters on the *XYZ* axes are in this video: 28K revolutions per minute (RPM); 130–140”/min; 0.160” step over; 0.0225 step down.

### 3.2. Different Materials: Operation Materials of CNC Milling Process

- Material Density of Wood to Milling Process Operation

In Figure 7a, using material wood samples by a CNC machine determines the milling process’ signal information on wood material with an automated CNC router machine.

- Material Density of Styrofoam to Milling Process Operation

In Figure 7b, using a sample of Styrofoam material by a CNC machine determines the milling process’ signal information on Styrofoam material with a CNC machine from DATRON foam cutter.

- Material Density of Plastic to Milling Process Operation

In Figure 7c, using plastic material samples by a CNC machine determines the milling process signal information on plastic materials. For CNC cutting machine parameters, this material is 1-flute, 1/8 Up spiral Endmill; super-PID controlled router at 10K revolutions per minute (RPM); 315+ inches per minute (IPM) rapids (10 mm lead ball screws on *X* and *Y*).

## 4. Results and Discussion

After the sound signal is received, the waveform parameters are determined as stable resonance information. Analysis of the realization of sound detection for each sample was applied with the LabVIEW program and re-plotted using the Origin Software [40], and the signals obtained after conducting 10 experiments were grouped with the K-means algorithm in the LabVIEW program and re-plotted using Excel.

### 4.1. Resonant Frequency Calibration with Do Re Mi Fa Sol Tone

**Figure 8:**
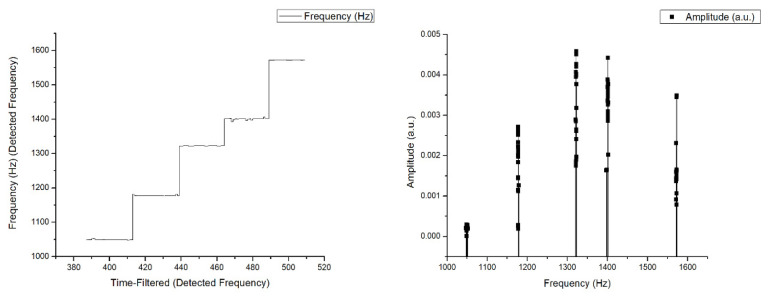
Experimental result for the frequency of the tone “Do Re Mi Fa Sol”.

The calibration simulation was carried out with Do Re Mi Fa Sol Tone played sequentially to see the resulting frequency range, then played back randomly 10 times, and always produced frequencies with the same range, “Do” at 10,050 Hz, “Re” at 1800 Hz, “Mi” at 1250 Hz, “Fa” at 1400 Hz, and “Sol” at 1570 Hz. This proves that the designed sound detection is consistent and stable. The results obtained are following the results of the frequency issued by each tone shown in Figure 8 as below:

### 4.2. Analysis for Operation Mode Process

**Figure 9:**
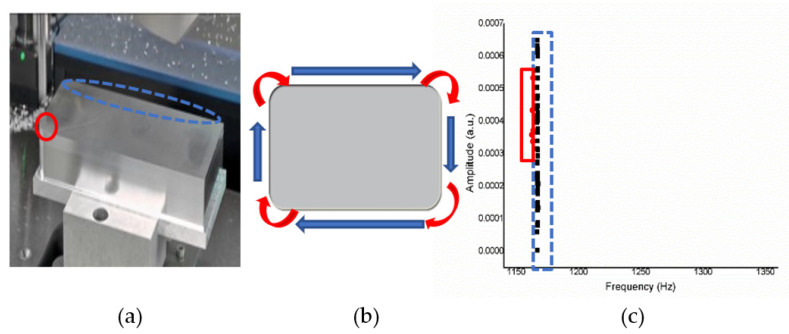
(**a**) Operation sketch, (**b**) trajectory of tool, (**c**) frequency of milling process for *XY* axes.

**Figure 10:**
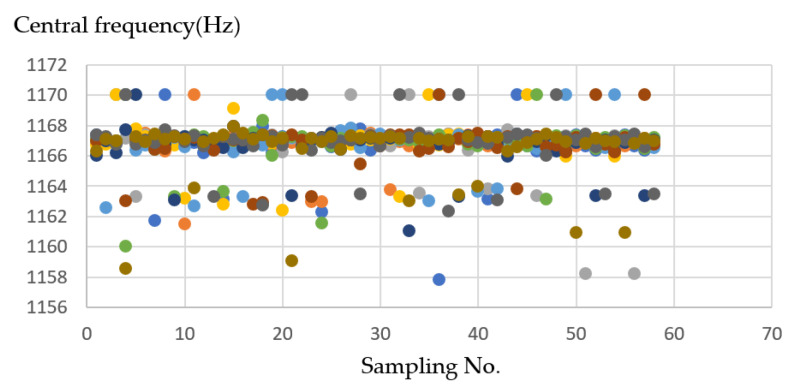
Ten sample frequency results from clustering by K-means for the *XY* axes.

**Figure 11:**
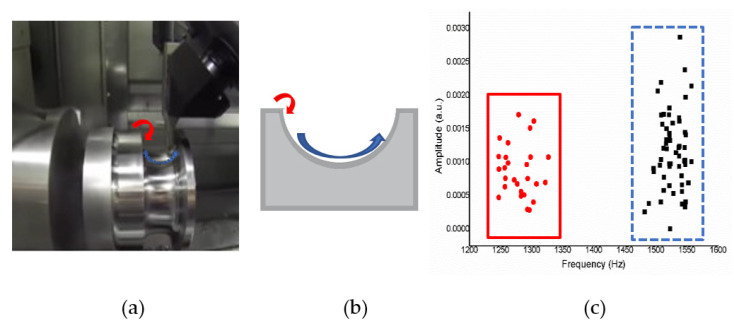
(**a**) Operation sketch, (**b**) trajectory of tool, (**c**) frequency of milling process for *XZ* axes.

**Figure 12:**
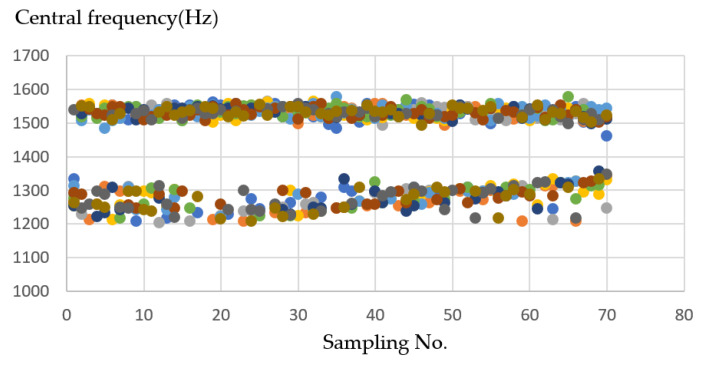
Ten sample frequency results from clustering by K-means for the *XZ* axes.

**Figure 13:**
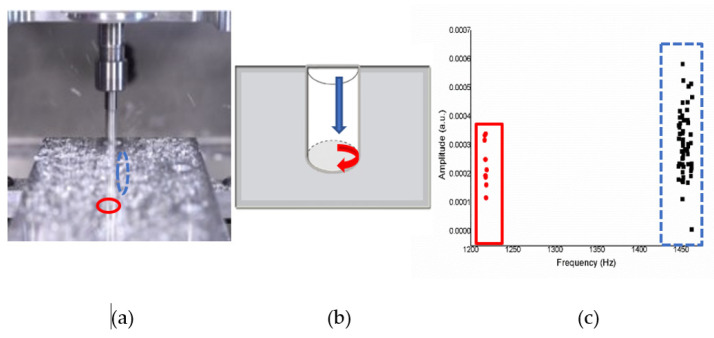
(**a**) Operation sketch, (**b**) trajectory of tool, (**c**) frequency of milling process for *XYZ* axes.

**Figure 14:**
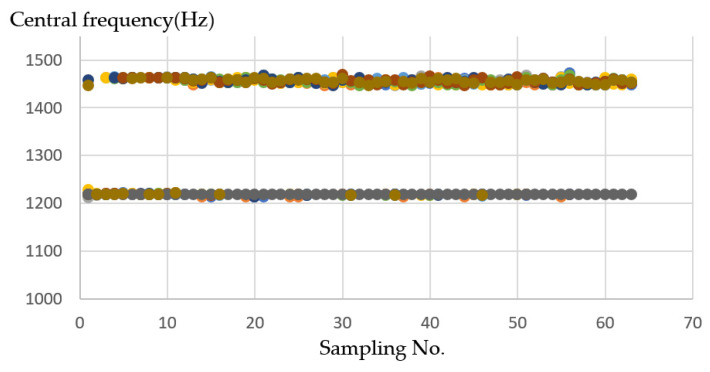
Ten sample frequency results from clustering by K-means for the *XYZ* axes.

Analysis for operation mode process using a CNC process formed with the *XY* axes shown in Figure 9 and Figure 10, *XZ* axes shown in Figure 11 and Figure 12, and *XYZ* axes shown in Figure 13 and Figure 14.

- *XY* Axes

Figure 9a shows an operation sketch of *XY* axes. When milling leads to shaping at corners (*X*) such as in Figure 9b, a frequency of around 1170 Hz is obtained, and when milling continues, the unidirectional process (*Y*) produces a frequency of 1157 Hz. Experiments will be carried out 10 times, and the results obtained will be of the same range. The frequency generated by the CNC milling process video can be obtained for voice changes, alternating between 1170 Hz and, then, 1157 Hz, as shown in Figure 9c.

On the *XY* axis shown in Figure 10, the frequency difference between the *X*-axes and the *Y*-axes are not significant, and the data similarity is high between 1157 and 1170 (Hz). The average is about 1165–1167 (Hz). Therefore, the iterative conversion will be more frequent when grouping. It needs to wait for the program to find a suitable group center as the final result, and the final result will be closer.

- *XZ* Axes

Figure 11a shows an operation sketch of *XZ* axes. When the cutting position started such as in Figure 11b and reached the high position (*X*), the octave frequency of 1200–1350 Hz went down. When the low position (*Z*) goes up to 1458–1560 Hz, the experiment was repeated 10 times, and the results obtained were in a similar range. The frequency according to the CNC milling process can be obtained from time to time. The alternating sound changes between 1200 and 1350 Hz then 1458 and 1560 Hz are shown in Figure 11c, and each actual sound change is only about one second.

In the *XZ* axes, the *X*-axes frequency is about 1200–1350 (Hz). The Z-axes is about 1458–1560 (Hz) on average, so after grouping, you can see in Figure 12 two separated clusters; one is the *X*-axes sound frequency that will be drilled at the beginning, and the other one is the *Z*-axes sound frequency that will be drilled later.

- *XYZ* Axes

Figure 13a shows an operation sketch of *XYZ* axes. When the milling touched the material’s surface of the trajectory of the tool such as in Figure 13b, the frequency obtained was around 1220 Hz, and when rotating down, it was around 1450–1470 Hz. The experiment was repeated 10 times. The frequency generated by the CNC milling process video can be obtained to sound changes, back and forth between 1220 Hz and, then, 1450–1470 Hz, as shown in Figure 13c.

In the *XYZ* axes, when the drill bit is drill down, the X-axes sound frequency will appear, so the amount of data obtained will be more, the frequency is about 1450–1470 (Hz). The frequency of the *Y* and *Z* axes is steadily located at about 1220 (Hz). Therefore, after grouping, it can be seen in Figure 14 that the numbers in the two clusters are different, but the cluster classification is precise.

### 4.3. Analysis for Different Material

**Figure 15:**
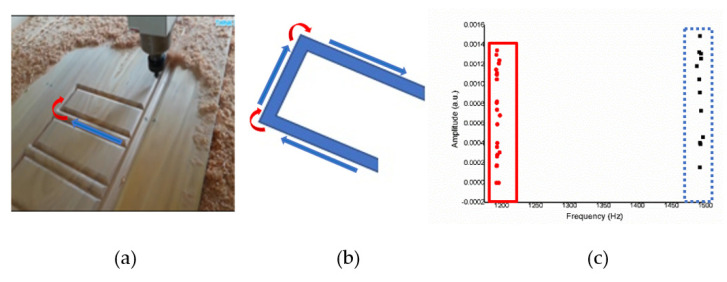
(**a**) Operation sketch, (**b**) trajectory of tool, (**c**) frequency of milling process for wood material.

**Figure 16:**
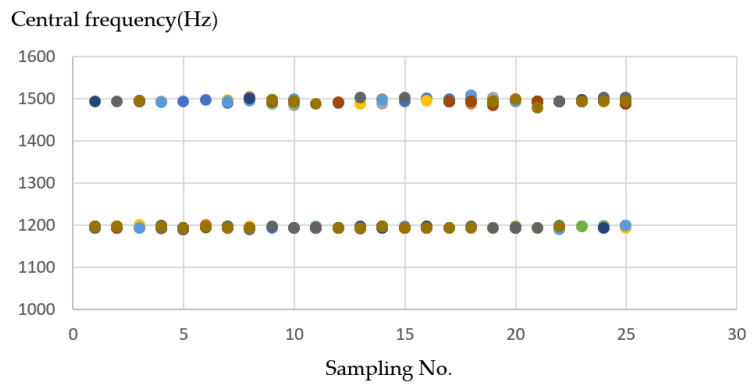
Ten sample frequency results from clustering by K-means for wood material.

**Figure 17:**
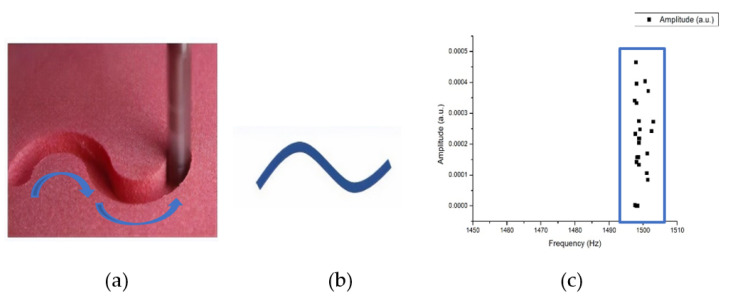
(**a**) Operation sketch, (**b**) trajectory of tool, (**c**) frequency of milling process for Styrofoam material.

**Figure 18:**
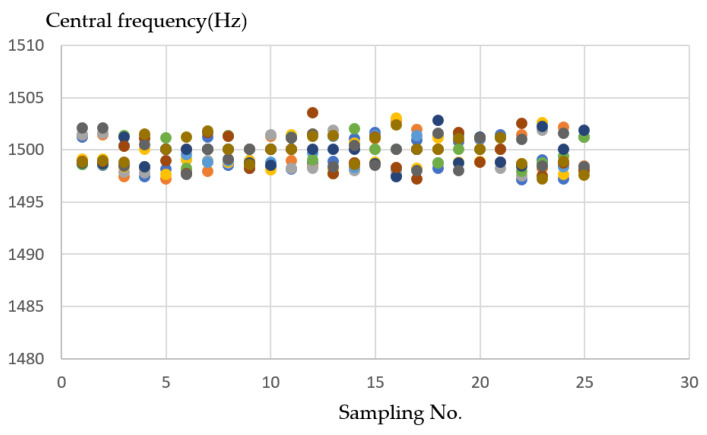
Ten sample frequency results from clustering by K-means for Styrofoam material.

**Figure 19:**
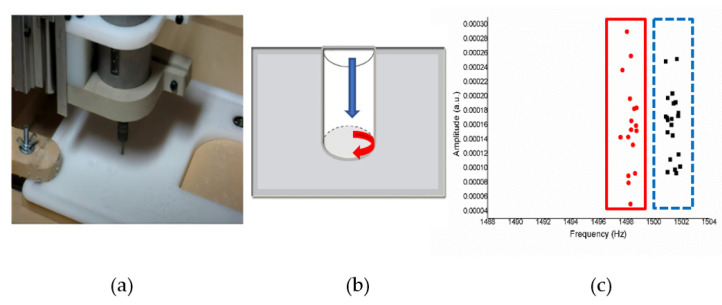
(**a**) Operation sketch, (**b**) trajectory of tool, (**c**) frequency of milling process for plastic material.

**Figure 20:**
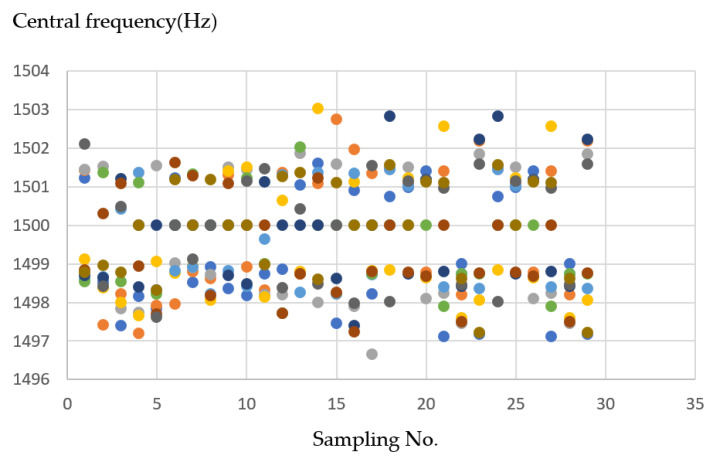
Ten sample frequency results from clustering by K-means for plastic material.

In simulations with different materials, the materials used are wood shown in Figure 15 and Figure 16. Styrofoam shown in Figure 17 and Figure 18, and plastic shown in Figure 19 and Figure 20.

- Simulation result with wood material.

Figure 15a shows an operation sketch for wood material. When the milling position reaches the start shown on the trajectory of a tool in Figure 15b, the octave frequency is around 1500 Hz; when the milling continues, it will be around 1200 Hz. The experiment was repeated 10 times. The alternating sound changes between 1200 Hz then 1500 Hz are shown in Figure 15c, and each actual sound change is only about one second.

Under the wood material, it can be seen in Figure 16 that when the milling machine drills a straight line and a right angle, the sound frequency changes significantly. When encountering a turning pitch, the frequency is considerably higher, about 1500 (Hz), while the linear frequency is stable at about 1200 (Hz). There are apparent divisions because the straight-line distance is longer, the drilling time is also longer, so the data obtained are relatively high.

- Simulation result with Styrofoam material.

For experiments on the Styrofoam material, the shape that will be formed by CNC milling is the S shape such as in the operation sketch in Figure 17a. When the tool started in the trajectory of a tool such as that in Figure 17b, the frequency of the milling process in Figure 17c is around 1495–1505 Hz. For the detection performed on the Styrofoam material, it records sound when performing the track without a load, so there is no distension signal for the Styrofoam material.

Styrofoam is relatively soft compared to other materials. In Figure 18 shows the sound frequency is relatively unchanged. When nothing is milling, the frequency is naturally 0, and the Styrofoam sound frequency will fall in the range of 1495~1505 (Hz), and the neatest and clear division can be seen from the grouping.

- Simulation result with plastic material.

Figure 19a shows an operation sketch for plastic material when the milling started the process shown on the trajectory of a tool in Figure 19b. When milling touches the material’s surface, the frequency is around 1495–1499 Hz, and when the milling process rotates, it forms a circle and the endpoint hole is 1501–1503 Hz, as shown in Figure 19c.

In plastic material, it can be seen in Figure 20, the frequency graph that the values are too close. Therefore, the cluster centers may be too close to each other, resulting in too many iterations and unstable results. However, there was a phenomenon in the past; the maximum number of iterations is set to 100, so the result of 100 iterations is the final result.

### 4.4. Results for Example if an Error Occurs

**Figure 21:**
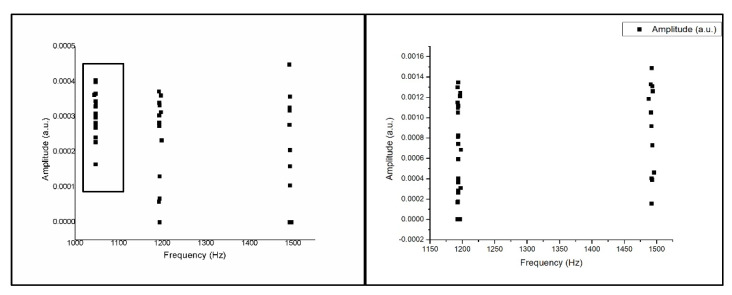
For example, if an error occurs and is normal.

The results for the example if an error occurs are obtained using one of the samples from the six videos, namely wood, in this experiment. We will combine the wood video as signal and “Do” sound as noise to prove the effectiveness of this monitoring system shown in the Figure 21.

As seen in the left picture, when there is a sound difference during the sound detection monitoring process, the result of the frequency that appears on the monitor does not match the sound rhythm when it is normal. So, we can find out by monitoring if there is a difference in the spectrum during the milling process on a CNC.

## 5. Conclusions

This study uses a sound detection program in LabVIEW as system integration. The experiment was carried out on the tone “Do Re Mi Fa Sol” to prove that the program was running well and obtained the same and stable frequency range for the calibration.

The conclusions are based on the results of real-time testing and the simulation of six sound videos of the CNC milling process with operation mode *XY*, *XZ*, *XYZ* axes and three different materials, namely wood, Styrofoam, and plastic.

The result of operation mode for the *XY* axes when milling leads to shaping at corners (*X*); a frequency of around 1170 Hz is obtained. When milling continues, the unidirectional process (*Y*) produces a frequency of 1157 Hz. For the *XZ* axes, when the cutting position reaches the high position (*X*), the octave 1200–1350 Hz frequency will go down, and the low position (*Y*) will go up to the frequency of 1458–1560 Hz. For the *XYZ* axes, when the milling touched the material’s surface, the frequency obtained was around 1220 Hz, and it reached the final point at 1450–1470 Hz.

The yield of different wood materials when the milling position reaches the start, the octave frequency is near 1500 Hz. As the milling continues, the frequency is near 1200 Hz. In the Styrofoam material, the result is in the form of a frequency near 1495–1505 Hz. For plastic material, when the milling touches the material’s surface, the frequency is close to 1495–1499 Hz, and when the milling process turns around to form a circle, the endpoint hole is 1501–1503 Hz.

In this test, it was found that the sound detection program was running well and when clustered using the K-means algorithm, the results were appropriate. Additionally, if there was a change in sound during the milling process or signal constraints, the operating point was linked to resonance frequency and amplitude to monitor the CNC production process in real time, so that if there is an error or vibration outside the process that occurs, it can be monitored. This system can be applied to various types of CNC milling processes, which work well for process data.

For further research, after reading several reference journals [41,42,43,44], it is possible to make an alarm notification during the milling process. If an error occurs during the milling process, it will be notified via an alarm, and this research can be continued by combining artificial intelligence to monitor more than one machines for one operator.

## Figures and Tables

**Table 1:** Parameters used in this experimental.

Parameters	Experiment Data in Simulation
Microphone (Realtek High Definition Audio)	Channel 1
Lower cut-off frequency [19], ω*_l_*	1000 (Hz)
Uppercut-off frequency, ω*_u_*	1700 (Hz)
Butterworth topology	Order (3)
Approximate frequency	1350 (Hz)

## References

[1] Wang P.-L., Tsai Y.-T. (2018). Numerical Analysis of CNC Milling Chatter Using Embedded Miniature MEMS Microphone Array System. Inventions.

[2] Sun P., Lu W., Hu H., Zhang Y., Chen M., Yan P. (2021). A Bayesian Approach to Predict Blast-Induced Damage of High Rock Slope Using Vibration and Sonic Data. Sensors.

[3] Jaber A.A., Bicker R. A Simulation of Non-stationary Signal Analysis Using Wavelet Transform Based on LabVIEW and Matlab. Proceedings of the 2014 European Modelling Symposium.

[4] Diana C., Ileana-Constanta R. Use of feature extraction algorithm in real-time echo detection application development. Proceedings of the 2013 E-Health and Bioengineering Conference (EHB).

[5] Tanuska P., Spendla L., Kebisek M., Duris R., Stremy M. (2021). Smart Anomaly Detection, and Prediction for Assembly Process Maintenance in Compliance with Industry 4.0. Sensors.

[6] Kuo S.-W., Raihany U., Peng C.-Y. Sound Detection of CNC Milling Machine by Embedded System. Proceedings of the 2020 International Symposium on Computer, Consumer and Control (IS3C).

[7] Shrenika R.M., Chikmath S.S., Kumar A.V.R., Divyashree Y.V., Swamy R.K. Non-contact Water Level Monitoring System Implemented Using LabVIEW and Arduino. Proceedings of the 2017 International Conference on Recent Advances in Electronics and Communication Technology (ICRAECT).

[8] Monteiro C.S., Raposo M., Ribeiro P.A., Silva S.O., Frazão O. (2021). Acoustic Optical Fiber Sensor Based on Graphene Oxide Membrane. Sensors.

[9] Kuralkar A.R. Design of Fast Fourier Transform Using Processing Element for Real-Valued Signal. Proceedings of the 2015 International Conference on Communications and Signal Processing (ICCSP).

[10] Kanungo T., Mount D.M., Netanyahu N.S., Piatko C.D., Silverman R., Wu A.Y. (2002). An efficient k-means clustering algorithm: Analysis and implementation. IEEE Trans. Pattern Anal. Mach. Intell..

[11] Shah S., Singh M. Comparison of a Time Efficient Modified K-mean Algorithm with K-Mean and K-Medoid Algorithm. Proceedings of the 2012 International Conference on Communication Systems and Network Technologies.

[12] Changalasetty S.B., Badawy A.S., Thota L.S., Ghribi W. Classification of moving vehicles using k-means clustering. Proceedings of the 2015 IEEE International Conference on Electrical, Computer and Communication Technologies (ICECCT).

[13] Altrabsheh B. (2010). Measurement and classification of heart and lung sound using LabVIEW for educational use. J. Med Eng. Technol..

[14] Józwik J. (2016). Identification and Monitoring of Noise Sources of CNC Machine Tools by Acoustic Holography Methods. Adv. Sci. Technol. Res. J..

[15] Józwik J., Wac-Włodarczyk A., Michałowska J., Kłoczko E.M. (2018). Monitoring of the Noise Emitted by Machine Tools in Industrial Conditions. J. Ecol. Eng..

[16] Rong H., Ramirez-Serrano A., Guan L., Gao Y. (2020). Image Object Extraction Based on Semantic Detection and Improved K-Means Algorithm. IEEE Access.

[17] Wu R., Xu Z., Zhang J., Zhang L. (2021). Robust Global Motion Estimation for Video Stabilization Based on Improved K-Means Clustering and Superpixel. Sensors.

[18] Aisyah S., Untari D., Wijanarko H. Design of Data Acquisition System of Environmental Parameter in Riau Islands using myRIO. Proceedings of the 2019 2nd International Conference on Applied Engineering (ICAE).

[19] Chen X., Shum P., Hu J.J. (2007). Special Control of the Cutoff Frequencies in a 2D Photonic Crystal Coupled-Cavity Waveguide. Opt. Commun..

[20] Agrawal N., Kumar A., Bajaj V., Singh G.K. (2019). Design of Bandpass and Bandstop Infinite Impulse Response Filters Using Fractional Derivative. IEEE Trans. Ind. Electron..

[21] Nam S., Lee B., Lee J. (2017). Theory for Pseudo-Butterworth Filter Response and Its Application to Bandwidth Tuning. IEEE Trans. Microw. Theory. Tech..

[22] Clark C.J., Silva C.P., Moulthrop A.A., Muha M.S. (2002). Power-amplifier characterization using a two-tone measurement technique. IEEE Trans. Microw. Theory Tech..

[23] Sowjanya K., Kumari B. (2013). Design and Performance Analysis of 32 And 64 Point FFT Using Radix-2 Algorithm. Int. J. Comput. Appl..

[24] Rafezi H., Akbari J., Behzad M. Tool Condition Monitoring based on sound and vibration analysis and wavelet packet decomposition. Proceedings of the 2012 8th International Symposium on Mechatronics and Its Applications.

[25] Banerjee S., Choudhary A., Pal S. Empirical evaluation of K-Means, Bisecting K-Means, Fuzzy C-Means, and Genetic K-Means clustering algorithms. Proceedings of the 2015 IEEE International WIE Conference on Electrical and Computer Engineering (WIECON-ECE).

[26] Mahmud S.M.H., Hossin M.A., Jahan H., Noori S.R.H., Bhuiyan T. CSV-ANNOTATE: Generate annotated tables from CSV file. Proceedings of the 2018 International Conference on Artificial Intelligence and Big Data (ICAIBD).

[27] Tewinkel G.C. (1959). Mathematical Basis of Analytic Aerotriangulation.

[28] Rochardjo H.S.B., Sakanegara B.G. Development of styrofoam cutter NC machine for intricate cutting path. Proceedings of the 2017 7th International Annual Engineering Seminar (InAES).

[29] Wu L., Ye Q., Yao L., Huang R., Wang J. The Data Processing System of CNC Wood-Working Milling Machine. Proceedings of the 2011 Second International Conference on Digital Manufacturing & Automation.

[30] Zhang H., Ai C., Zhao F., Ze X. The CNC development of the sawing and milling machining center for plastic door and window PVC profile based on Fanuc0. Proceedings of the 2008 7th World Congress on Intelligent Control and Automation.

[31] Datron A.G. DATRON CNC Project: Packaging Industry [Video]. https://youtu.be/qQi3EN_wR80.

[32] Gobekoglu H. EdgecamWaveform on Okuma Multus U3000 [Video]. https://youtu.be/gRZMiyjv-S4.

[33] Bantam Tools What Does it Sound like to Machine Aluminum? [Video]. https://youtu.be/lwyqQCDKY_g.

[34] Liu Y., Zhang H., Wang X. (2017). Analysis on Influence of Perpendicularity Error of Five-Axes NC Machine Tool Error Modeling Accuracy and Complexity. Procedia Eng..

[35] Liu Y., Wan M., Xing W.-J., Zhang W.-H. (2018). Identification of position-independent geometric errors of rotary axes for five-axes machine tools with structural restrictions. Robot. Comput. Integr. Manuf..

[36] Xie Y., Li Y., Cheung C.F., Zhu Z., Chen X. (2020). Design and analysis of a novel compact XYZ parallel precision positioning stage. Microsyst. Technol..

[37] THink Media 79 Giant Wooden Door Design with Automated CNC Router—Amazing Modern Woodworking Machine [Video]. https://youtu.be/5Ua1_vdUnV0.

[38] Datron A.G. DATRON Schaumstofffräser-Hochgeschwindigkeits-Fräsen von PU-Schaum [Video]. https://youtu.be/q_36kSEvWdM.

[39] Walky My CNC Cutting Some Plastic [Video]. https://youtu.be/vurdVkGl05w.

[40] Godfrey M.W., Zou L. (2005). Using origin analysis to detect merging and splitting of source code entities. IEEE Trans. Softw. Eng..

[41] Zhang X., Xu Q. Design and analysis of an in-plane flexure XYZ micro/nano-positioning stage. Proceedings of the 2016 International Conference on Advanced Robotics and Mechatronics (ICARM).

[42] Nilsson H., Sidén J., Gulliksson M. An incontinence alarm solution utilizing RFID based sensor technology. Proceedings of the 2011 IEEE International Conference on RFID-Technologies and Applications.

[43] Xiao F., Miao Q., Xie X., Sun L., Wang R. (2018). Indoor Anti-Collision Alarm System Based on Wearable Internet of Things for Smart Healthcare. IEEE Commun. Mag..

[44] Deshpande S.M., Ainapure B. An Intelligent Virtual Machine Monitoring System Using KVM for Reliable and Secure Environment in Cloud. Proceedings of the 2016 IEEE International Conference on Advances in Electronics, Communication and Computer Technology (ICAECCT).

